# Supplementary material for: Variation in appendages in early Cambrian bradoriids reveals a wide range of body plans in stem-euarthropods
Source: Commun Biol. 2019 Sep 3;2:329. doi: 10.1038/s42003-019-0573-5 (PMC6722085; doi:10.1038/s42003-019-0573-5)
Supplement: Supplementary file 1 — Supplementary information [file 42003_2019_573_MOESM1_ESM.docx]

**Variation in appendages in early Cambrian bradoriids reveals a wide range of body plans in stem-euarthropods**

**Supplementary Figures**

**
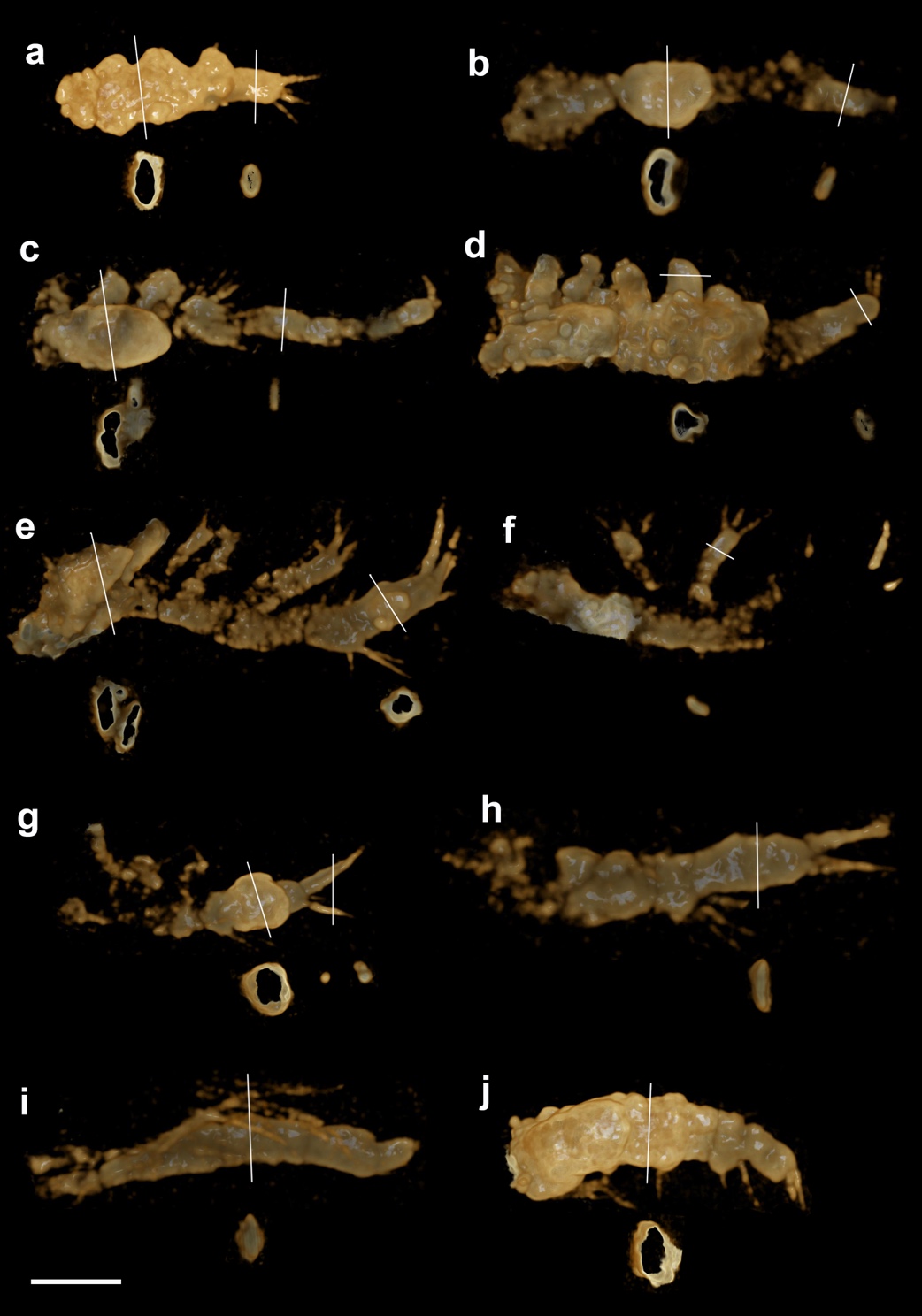
**

**Supplementary Figure 1** **| Taphonomy of *Kunyangella cheni* Huo, 1965.** Right lateral views of various appendages and their cross sections (white lines show position of cross sections) of YKLP 16232, showing different degrees of compression. **a**, right appendage 1. **b**, right appendage 2. **c**, right appendage 3. **d**, right appendage 4. **e**, right appendage 5. **f**, right appendage 6. **g**, right appendage 7. **h**, right appendage 8. **i**, right appendage 9. **j**, right appendage 10. In appendages 1-5 and 10 anatomical details appear faithfully preserved, whereas in appendages 6-9 the anatomy is incompletely preserved. Scale bar: **a** 220 µm; **b** 130 µm; **c** 170 µm; **d** 180 µm; **e** 210 µm; **f** 190 µm; **g** 200 µm; **h** 140 µm; **i** 170 µm; **j** 180 µm.

**
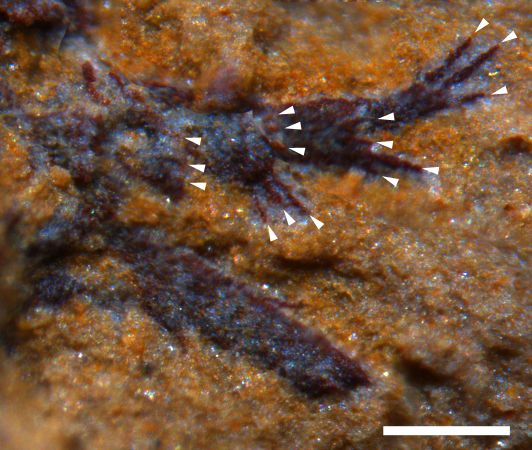
**

**Supplementary Figure 2 | Antenna (a1) of *Indiana* sp.** Several terminal setae are visible on podomeres 2-5, some of which are not visible in the CT image of Fig. 2K (main text). Scale bar: 200 µm. The counterpart view of this specimen is depicted in Fig. 2K.

**Supplementary Notes**

Systematic Palaeontology

Euarthropoda Lankester, 1904^1^

?Order Bradoriida Raymond, 1935^2^

*Remarks. Kunmingella douvillei, Kunyangella cheni* and *Indiana* sp. are conventionally assigned to the Bradoriida on carapace morphology^3^. However, our micro-CT study demonstrates significant differences in limb morphology between the three species. The nominate genus of the Bradoriida, *Bradoria* Matthew,1899^4^, is known only from its carapace, and therefore we cannot objectively assess the affinities of the Chinese material to it. Given this, and our phylogenetic analysis (main text), our assignment of the three Chinese species to Bradoriida is tentative.

Family Kunmingellidae Huo & Shu, 1985^5^

Genus *Kunmingella* Huo, 1956^6^

*Kunmingella douvillei* (Mansuy, 1912)^7^ (main text Fig. 1a-i)

1912 *Bradoria douvillei* sp. nov.; Mansuy^7^, p. 22, pl. 1, fig. 8.

1996 *Kunmingella maotianshanensis* Huo & Shu *in* Huo *et al*. 1983; Hou *et al*.^8^, p. 1132, figs 3-5.

1999 *Kunmingella maotianshanensis* Huo & Shu *in* Huo *et al*.1983; Shu *et al*.^9^, figs 1-6, 10.

2002 *Kunmingella douvillei* (Mansuy, 1912); Hou *et al*.^3^, p. 359, figs 11a-k, 12a-j, 13a-h (q.v. for full synonymy).

2004 *Kunmingella douvillei* (Mansuy, 1912); Hou *et al*.^10^, p. 114, fig. 16.13-c.

2010 *Kunmingella douvillei* (Mansuy, 1912); Hou *et al*.^11^, p. 1836, figs 1a, d-o, 2.

2014 *Kunmingella douvillei* (Mansuy, 1912); Duan *et al*.^12^, figs1a-h, 2, 3a-c, 4a-d, 5.

2017 *Kunmingella douvillei* (Mansuy, 1912); Hou *et al*.^13^, p. 228, figs 3.3, 5.1, 20.54, 20.55a-d.

*Syntypes.* Carapaces and valves on a slab of mudstone^7^. Kebaocun section, Yiliang County, Yunnan. Repository unknown.

*Material.* Thousands of specimens^11,13^, recorded from Yunnan, Shaanxi and Sichuan provinces.

*X-rayed material*. YKLP 16233 (1.85 mm long) and YKLP 16235 (2.53 mm long) from Mafang, Haikou.

*Diagnosis*. *Kunmingella* with valves lacking a posteroventral spine. Twelve pairs of appendages, including an antenna with 5 podomeres; appendages 2-5 each with an endopod bearing a double row of endites; and a long uniramous 11th appendage.

*Description*. Postplete, bivalved carapace (3 to 5.24 mm long; after^3^) with a tapered posterior lobe, an anterodorsal node and a narrow adventral structure extending between the cardinal corners. Valves smooth. Twelve pairs of appendages, 5 forwardly projecting pairs (interpreted as the head region) and 7 posteriorly projecting pairs in the trunk. A pair of lateral eyes occurs just anterior to the origin of the first appendage. A basipod is not evident in any of the appendages.

The antenna is uniramous, consists of at least 5 podomeres and projects forward beyond the carapace margin (main text Fig. 1a-c). Four of the podomeres have a single seta distally on the outer margin and an endite bearing at least 3 setae distally on the inner margin (only partially visible on podomere 1); the terminal podomere bears at least 6 setae (main text Figs 1c, 3a). Appendages 4 and 5 are biramous (main text Fig. 1a,b,f). The endopod consists of at least 9 podomeres, each having 2 elongate endites (collectively forming 2 rows) each with at least a tiny seta terminally (main text Figs 1d, f, 3a). The exopod is lamella-like, paddle-shaped (main text Figs 1f, 3a) and bears marginal setae^11^. The same kind of endopod characterises appendages 2 and 3, which probably also have a paddle-shaped exopod though this is not evident.

Appendages 6-10 are biramous (main text Figs 1a,b, 3a). The endopod has up to 8 podomeres as seen in appendage 6 (main text Fig. 1g), each bearing a single elongate endite with a terminal seta. Six podomeres are evident in appendage 7, and at least 4 in appendage 8. The exopod is paddle-shaped with marginal setae and a longer terminal seta (main text Figs 1g, 3a). Appendage 9 is poorly preserved, but it shows several proximal podomeres. At least 8 podomeres are evident in appendage 10, which bears the remnants of a paddle-shaped exopod (main text Fig. 1b). The 11th appendage is long and trails posteriorly beyond the carapace (main text Fig. 1i). It consists of at least 6 elongate podomeres each with a weak endite bearing a seta(e) on the inner margin (though not visible in Fig. 1i, main text); the distal-most podomere bears a long terminal seta. The 12th appendage is short, consists of 4 podomeres, and is gradually tapered (main text Figs 1i, 3a). Podomeres 1-3 each bear 2 short, stiff setae, 1 on the outer margin and 1 on the inner margin respectively. At least 6 setae occur on the distal-most podomere (see^11^, fig. 1h,i). At the end of the trunk there is a short triangular tailpiece (main text Figs 1i, 3a).

*Discussion*. Previous analyses^11^, lacking micro-CT examination, recognised only 10 pairs of appendages in *Km. douvillei*. Our material confirms previous reports (e.g.^9,12^) that *Km. douvillei* had an egg brooding strategy, with at least 25 tiny eggs in a single individual (main text Fig. 1a,f). There is no evidence of eggs in the two other bradoriid taxa described here. Fossil eggs of invertebrates are rare, and most supposed such eggs occur isolated from the assumed parent, with just a few examples of putative *in situ* embryos^14,15^, most of which are documented in early Palaeozoic ostracods. Duan *et al*.^12^ record that *Km. douvillei* carried its eggs on the endopods of its posterior trunk appendages and also opined that the species possibly had a K-reproductive strategy. In contrast, in our egg-bearing specimen of *Km. douvillei* the eggs occur mid-valve, nestled in the region of the lobal structure. Similar brooding strategies are widespread in ostracods, which have used a range of specialized carapace structures to accommodate the eggs (see^16^ for a review). The eggs and apparent brooding strategy of *Km. douvillei* probably indicate female gender, though it is not certain. Male brood care is also a possibility, as is known, for example, in pycnogonid euarthropods^17^.

Family Comptalutitdae Öpik, 1968^18^

Genus *Kunyangella* Huo, 1965^19^

*Kunyangella cheni* Huo, 1965^19^ (main text Fig. 2a-i, m-o)

1965 *Kunyangella cheni* sp. nov.; Huo^19^, p. 293, pl. 1, fig. 6.

2002 *Kunyangella cheni* Huo, 1965; Hou *et al*.^3^, p. 387, figs 20d – k, 21a (q.v. for full synonymy).

2010 *Kunyangella cheni* Huo, 1965; Hou *et al*.^11^, p. 1838, fig. 1b, c.

2017 *Kunyangella cheni* Huo, 1965; Hou *et al*.^13^, p. 230, fig. 20.56, 20.57.

*Holotype*. Juvenile(?) left valve external mould, Northwest University, Xian, China, no. 0006, Jinning County, Yunnan^19^.

*Material*. Over 100 specimens from several localities in Yunnan^3^.

*X-rayed material*. YKLP 16232 (2.82 mm long) from Mafang, Haikou, and YKLP 16236 (2.89 mm long) from Erjie, Jinning.

*Diagnosis.* For the monotypic genus (amended from^11^). Carapace with a single, bulbous, elongate to arcuate mid-dorsal node. Thirteen pairs of appendages, including an antenna with a minimum of 9 podomeres. At least 8 of the post-antennal appendages bear short ovate exopods and slender endopods bearing up to 10 podomeres. There is a uniramous terminal appendage (= 10^th^ appendage in YKLP 16232 and 13^th^ appendage in YKLP 16236) comprising at least 9 podomeres.

*Description*. Carapace postplete, adults up to 2.89 mm long. Lateral valve outline with a posterodorsal curve and weak anterodorsal curve. Lateral outline between these curves and the cardinal corners is fairly straight to weakly concave, ventrally is rounded and convex to strongly convex. Bulbous mid-dorsal node is elongate to arcuate, oriented sub-parallel to the dorsal margin. Weak, V-shaped depression developed anterior of node, may demarcate a very weak anterodorsal node. Adventral ridge entire between cardinal corners, weakly developed, demarcated from the lateral valve surface by a furrow. Valves smooth.

There are 13 pairs of appendages. No indication of eyes. The antenna is uniramous, stout and comprises at least 9 podomeres (main text Fig. 2f) that gradually narrow along the axis of the limb towards the distal end. At least 8 post-antennal appendages are biramous, bearing short ovate exopods, as seen on appendages 2 to 6 of YKLP 16232 (main text Figs 2a,d,e,g, 3b) and appendages 7 and 8 on YKLP 16236 (main text Fig. 2c), that extend to the base of the 4th podomere of the endopod. The endopod of the 2nd appendage comprises several slender podomeres lacking endites (main text Fig. 2d). The 3rd appendage has an endopod with 4 proximal podomeres each bearing an endite, and 5 distal slender podomeres that lack endites; the terminal podomere bears 2 short setae (main text Figs 2e, 3b). The 4th appendage bears 7 stout podomeres proximally, each with a single long endite, and 3 slender podomeres distally that lack endites; the terminal podomere bears 2 setae (main text Figs 2g, 3b). The 5th appendage (considered to be the 1st trunk appendage) bears 8 slender podomeres, the first 4 bearing elongate endites, the terminal 4 lacking endites; the terminal podomere bears 4 setae, at least 2 of which are pronounced and resemble a ‘claw’ (main text Fig. 2h); the endites of the proximal podomeres bear up to 3 distal setae. The posterior appendages are more poorly preserved, but in YKLP 16232 (main text Fig. 2a) the endopods of appendages 6 to 8 appear to have a similar morphology to appendage 5, with elongate endites bearing at least 3 terminal setae (visible in appendage 6), and a terminal podomere in the endopod with at least 2 pronounced ‘claw-like’ setae (visible in appendages 6-8; see main text Fig. 2a,m,n). In addition, the 6th appendage of YKLP 16232 shows a short ovate exopod, and the 7^th^ and 8^th^ appendage of YKLP 16236 show exopods of similar morphology (main text Fig. 2a,c). The posterior-most appendage (13^th^ in YKLP 16236, 10^th^ in YKLP 16232) is stout and uniramous and comprises 9 podomeres with short endites that bear setae (main text Fig. 2o) including a narrow terminal podomere lacking an endite but with at least 2 terminal setae.

*Discussion*. The two *Ky. cheni* that have been micro-CT scanned are of similar size but bear a different number of appendages; YKLP 16232 has 10 pairs and YKLP 16236 has 13 pairs. *Kunmingella douvillei* is known to add appendages (and segments) during ontogeny^11^, with 3 appendages (and presumably 3 segments) added between growth stage 4 (*sensu*^20^) and the adult stage. In the case of *Ky. cheni* we do not know whether the difference between the specimens is ontogenetic, the result of sampling populations with different overall size ranges, or taphonomic, some appendages being lost in YKLP 16232.

Family Bradoriidae Matthew, 1902^21^

Genus *Indiana* Matthew, 1902^21^

*Indiana* sp. (main text Fig. 2j-l)

*X-rayed material*. One specimen with appendages, YKLP 16231 (2.09 mm long), Jianshan, Haikou.

*Description*. Carapace elongate, ovoid, 2.09 mm long, greatest valve height just anterior of mid-length. Dorsal margin gently arched as preserved. Valves smooth and non-lobate, adventral structure absent. Twelve pairs of appendages. No indication of eyes.

Antenna stout, uniramous, and consisting of at least 5 podomeres, 4 of which bear a single short endite, podomeres 2 to 4 show short terminal setae (not visible in podomere 1; see supplementary Fig. 2), whilst the terminal podomere is narrow, elongate and bears 3 terminal setae (main text Figs 2k, 3c). Appendages 2 and 3 show at least 6 podomeres with up to 3 very long and forwards directed setae on the proximal part of the 4th podomere (as visible) of appendage 2; the terminal podomere of appendage 3 bears 3 terminal setae. Podomeres 1 to 3 (as visible) of appendages 2 and 3 may have born endites, but if so they are not preserved or have broken off, as evidenced by the ragged nature of the appendage margins (main text Fig. 2j). Appendages 4 to 10 display at least 8 podomeres, the terminal 3 podomeres of each endopod lacking endites, whilst the proximal 5 each bear a single elongate ‘castellate’ endite (main text Figs 2l, 3c) with up to 4 distal setae. The terminal podomere of limbs 4 to 10 have up to 3 elongate forwards-directed stout setae (main text Figs 2l, 3c), whilst elongate forward projecting setae like those on podomere 4 of appendage 2 are also visible on appendage 4. Posterior to appendage 10 are two further appendages that are incompletely preserved (main text Fig. 2j). Exopods of appendages 2-12 are not evident. A basipod is not evident in any of the appendages.

*Discussion*. The non-lobate, elongate ovate carapace allies this bradoriid with *Indiana*, a genus represented in the Cambrian of North America, Scandinavia and Britain^22^. In the specimen at hand the carapace is distorted, and the arched dorsal margin may partly be a function of preservation.

We interpret the post-antennal appendages of *Indiana* sp. to be essentially uniform, and characterized by 3 terminal podomeres lacking endites, the terminal podomere bearing 3 setae, whilst the more proximal podomeres bear a single elongate castellate endite (main text Fig. 3).

**Supplementary References**

1. Lankester, E.R. The structure and classification of Arthropoda. *Quarterly Journal of Microscopical Science* **47**, 523–582 (1904).

2. Raymond, P.E. *Leonchoila* and other Mid-Cambrian Arthropoda. *Bull. Mus. Comp. Zool. Harvard Univ*. **76**, 205–230 (1935).

3. Hou, X., Siveter, D.J., Williams, M. & Feng, X. A monograph of bradoriid arthropods from the Lower Cambrian of SW China. *Trans R. Soc. Edinb. Earth Sci.* **92** (for 2001), 347–409 (2002).

4. Matthew G.F. Preliminary notice of the Etcheminian Fauna of Cape Breton. *Bull. Nat. Hist. Soc. New Brunswick* **4**, 198–208 (1899).

5. Huo, S. & Shu, D. *Cambrian Bradoriida of south China*, pp. 251. Xian, China: Northwest University Press (1985).

6. Huo, S. Brief notes on Lower Cambrian Archaeostraca from Shensi and Yunnan. *Acta Palaeontol. Sin.* **4**, 425–445 (1956).

7. Mansuy, H. Pt. 2, Paléontologie. In: Deprat, J. & Mansuy, H., *Etude géologique du Yun-Nan oriental. Mémoires du service géologquie de l'Indochine*, **1**, 146 pp, 7 pls (1912).

8. Hou, X., Siveter, D.J., Williams, M., Walossek, D. & Bergström, J. Appendages of the arthropod *Kunmingella* from the early Cambrian of China: its bearing on the systematic position of the Bradoriida and the fossil record of the Ostracoda. *Phil. Trans R. Soc. B* **351**, 1131–1145 (1996).

9. Shu, D., Vannier, J., Luo, H., Chen, L., Zhang, X. & Hu, S. 1999 Anatomy and lifestyle of *Kunmingella* (Arthropoda, Bradoriida) from the Chengjiang fossil Lagerstätte (Lower Cambrian, Southwest China). *Lethaia* **32**, 279–298 (1999).

10. Hou, X. *et al*. *The Cambrian Fossils of Chengjiang, China: The Flowering of Early Animal Life*. Oxford: Blackwell Publishing (2004).

11. Hou, X., Williams, M., Siveter, D.J., Aldridge, R.J. & Sansom, R.S. Soft-part anatomy of the Early Cambrian bivalved arthropods *Kunyangella* and *Kunmingella*: significance for the phylogenetic relationships of Bradoriida. *Proc. R. Soc. B* **277**, 1835–1841 (2010).

12. Duan, Y., Han, J., Fu, D., Zhang, X., Yang, X., Komiya, T. & Shu, D. Reproductive strategy of the bradoriid arthropod *Kunmingella douvillei* from the Lower Cambrian Chengjiang Lagerstätte, South China. *Gondwana Research* **25**, 983–990 (2014).

13. Hou X. *et al*. *The Cambrian Fossils of Chengjiang, China: The Flowering of Early Animal Life*, 2nd Edition. Oxford: Wiley Blackwell (2017).

14. Siveter, D.J., Siveter, D.J., Sutton, M.D. & Briggs, D.E.G. Brood care in a Silurian ostracod. *Proc. R. Soc. B*, **274**, 465–469 (2007).

15. Siveter, D.J., Tanaka, G., Farrell, C.Ú., Martin, M.J., Siveter, D.J. & Briggs, D.E.G. Exceptionally preserved 450 million-year-old Ordovician ostracods with brood care. *Curr. Biol.* **24**, 801–806 (2014).

16. Ozawa, H. The history of sexual dimorphism in Ostracoda (Arthropoda, Crustacea) since the Palaeozoic. Chapter 4. *In* Moriyama, H. (ed.) *Sexual Dimorphism*. Intech Open (2013).

17. Requena, G.S., Munguía-Steyer, R. & Machado, G. Paternal care and sexual selection in arthropods. Chapter 8. pp. 201–223. In Macedo, R.H. & Machado, G. (eds) *Sexual selection. Perspectives and models from the* *neotropics*. Elsevier, Academic Press (2014).

18. Öpik A.A. Ordian (Cambrian) Crustacea Bradoriida of Australia. *Bull. Bur. Miner. Resour. Geol. Geophys.* **103**, 1–46 (1968).

19. Huo S. Additional notes on Lower Cambrian Archaeostraca from Shensi and Yunnan. *Acta Palaeontol. Sin*. **13**, 291–307 (1965).

20. Zhang, X. Phosphatized bradoriids (Arthropoda) from the Cambrian of China. *Palaeontogr. Abteilung* **A281**, 93–173 (2007).

21. Matthew, G.F. Ostracoda of the basal Cambrian rocks in Cape Breton. *Canadian Record of Science* **8**, 437–470 (1902).

22. Williams, M., Siveter, D.J., Popov, L.E. & Vannier, J.M.C. Biogeography and affinities of the bradoriid arthropods: cosmopolitan microbenthos of the Cambrian seas. *Palaeogeogr. Palaeoclimatol. Palaeoecol*. **248**, 202–232 (2007).
