# Supplementary material for: Variation in appendages in early Cambrian bradoriids reveals a wide range of body plans in stem-euarthropods
Source: Commun Biol. 2019 Sep 3;2:329. doi: 10.1038/s42003-019-0573-5 (PMC6722085; doi:10.1038/s42003-019-0573-5)
Supplement: Supplementary file 2 — Reporting Summary [file 42003_2019_573_MOESM2_ESM.pdf]

nature research

Corresponding author(s): Yu Liu, Xianguang Hou

Last updated by author(s): Jul 24, 2019

# Reporting Summary

Nature Research wishes to improve the reproducibility of the work that we publish. This form provides structure for consistency and transparency in reporting. For further information on Nature Research policies, see Authors & Referees and the Editorial Policy Checklist.

## Statistics

For all statistical analyses, confirm that the following items are present in the figure legend, table legend, main text, or Methods section.

| n/a | Confirmed | |
|---|---|---|
| ☒ | ☐ | The exact sample size (*n*) for each experimental group/condition, given as a discrete number and unit of measurement |
| ☒ | ☐ | A statement on whether measurements were taken from distinct samples or whether the same sample was measured repeatedly |
| ☒ | ☐ | The statistical test(s) used AND whether they are one- or two-sided<br>*Only common tests should be described solely by name; describe more complex techniques in the Methods section.* |
| ☒ | ☐ | A description of all covariates tested |
| ☒ | ☐ | A description of any assumptions or corrections, such as tests of normality and adjustment for multiple comparisons |
| ☒ | ☐ | A full description of the statistical parameters including central tendency (e.g. means) or other basic estimates (e.g. regression coefficient) AND variation (e.g. standard deviation) or associated estimates of uncertainty (e.g. confidence intervals) |
| ☒ | ☐ | For null hypothesis testing, the test statistic (e.g. *F*, *t*, *r*) with confidence intervals, effect sizes, degrees of freedom and *P* value noted<br>*Give P values as exact values whenever suitable.* |
| ☒ | ☐ | For Bayesian analysis, information on the choice of priors and Markov chain Monte Carlo settings |
| ☒ | ☐ | For hierarchical and complex designs, identification of the appropriate level for tests and full reporting of outcomes |
| ☒ | ☐ | Estimates of effect sizes (e.g. Cohen's *d*, Pearson's *r*), indicating how they were calculated |

*Our web collection on statistics for biologists contains articles on many of the points above.*

## Software and code

Policy information about availability of computer code

| | |
|---|---|
| Data collection | No software was used to collect the data. |
| Data analysis | We used the software Drishti (Versions 2.4 and 2.6.4) to process the micro-CT data and to generate 3-D models of the fossils. |

For manuscripts utilizing custom algorithms or software that are central to the research but not yet described in published literature, software must be made available to editors/reviewers. We strongly encourage code deposition in a community repository (e.g. GitHub). See the Nature Research guidelines for submitting code & software for further information.

## Data

Policy information about availability of data

All manuscripts must include a data availability statement. This statement should provide the following information, where applicable:

- Accession codes, unique identifiers, or web links for publicly available datasets
- A list of figures that have associated raw data
- A description of any restrictions on data availability

Data are available from the authors on request. Fossil specimens are deposited at the Yunnan Key Laboratory for Palaeobiology (YKLP), Yunnan University, China, with the accession numbers: YKLP 16231, 16232, 16233, 16235, 16236. For the phylogenetic analysis depicted in Figure 4, the data matrix and associated search commands are available via MorphoBank, project 3499 (http://morphobank.org/permalink/?P3499)

# Field-specific reporting

Please select the one below that is the best fit for your research. If you are not sure, read the appropriate sections before making your selection.

☐ Life sciences ☐ Behavioural & social sciences ☒ Ecological, evolutionary & environmental sciences

For a reference copy of the document with all sections, see nature.com/documents/nr-reporting-summary-flat.pdf

# Ecological, evolutionary & environmental sciences study design

All studies must disclose on these points even when the disclosure is negative.

| | |
|---|---|
| Study description | Analysis of three Cambrian bradoriid arthropod species with micro-CT identifies a markedly greater range of body plans than previously suspected. |
| Research sample | Five bradoriid fossils were analysed with micro-CT. |
| Sampling strategy | The bradoriids were collected from the Yu'anshan Member, Chiungchussu Formation, Eoredlichia-Wutingaspis trilobite biozone, Cambrian Series 2, Stage 3, Yunnan Province, China. Two specimens of Km. douvillei (YKLP 16233, YKLP 16235) were collected from Mafang, Haikou, Kunming; one specimen of Ky. cheni (YKLP 16232) is preserved on the same rock slab with Km. douvillei YKLP 16233. A second Ky. cheni (YKLP 16236) was collected from Erjie, Jinning, Kunming. The specimen of Indiana sp. (YKLP 16231) was collected from Mt. Jianshan, Haikou. These specimens all have preserved anatomy, they are exceptionally rare, and are identified only intermittently from intensive collecting over several years (in this case 16 years). |
| Data collection | Field collecting by three members of the Yunnan Key Laboratory for Palaeobiology team, namely Hou Xianguang, Dayou Zhai and Yu Liu |
| Timing and spatial scale | Over the period from November 1999 to September 2015 |
| Data exclusions | No data exclusions. Fossils with soft anatomy are very rare, and all such specimens were included in the study. |
| Reproducibility | All fossil data are reproducible (specimens are deposited in the museum collection of the YKLP. Our phylogenetic data are provided online. For the phylogenetic analysis, the data matrix and associated search commands are available via MorphoBank, project 3499 (http://morphobank.org/permalink/?P3499) |
| Randomization | This is not relevant to this study, which is documenting the anatomy of three species of Cambrian bradoriid arthropods |
| Blinding | This is not relevant to this study, which is documenting the anatomy of three species of Cambrian bradoriid arthropods |

Did the study involve field work? ☒ Yes ☐ No

## Field work, collection and transport

| | |
|---|---|
| Field conditions | Subtropical climate in central Yunnan Province |
| Location | The bradoriids were collected from the Yu'anshan Member, Chiungchussu Formation, Eoredlichia-Wutingaspis trilobite biozone, Cambrian Series 2, Stage 3, Yunnan Province, China. Two specimens of Km. douvillei (YKLP 16233, YKLP 16235) were collected from Mafang, Haikou, Kunming; one specimen of Ky. cheni (YKLP 16232) is preserved on the same rock slab with Km. douvillei YKLP 16233. A second Ky. cheni (YKLP 16236) was collected from Erjie, Jinning, Kunming. The specimen of Indiana sp. (YKLP 16231) was collected from Mt. Jianshan, Haikou. |
| Access and import/export | Not relevant, these are fossil samples.<br>All permissions from local government where received |
| Disturbance | No disturbance, natural rock outcrops or existing quarries |

# Reporting for specific materials, systems and methods

We require information from authors about some types of materials, experimental systems and methods used in many studies. Here, indicate whether each material, system or method listed is relevant to your study. If you are not sure if a list item applies to your research, read the appropriate section before selecting a response.

## Materials & experimental systems

| n/a | Involved in the study | |
|---|---|---|
| ☒ | ☐ | Antibodies |
| ☒ | ☐ | Eukaryotic cell lines |
| ☐ | ☒ | Palaeontology |
| ☒ | ☐ | Animals and other organisms |
| ☒ | ☐ | Human research participants |
| ☒ | ☐ | Clinical data |

## Methods

| n/a | Involved in the study | |
|---|---|---|
| ☒ | ☐ | ChIP-seq |
| ☒ | ☐ | Flow cytometry |
| ☒ | ☐ | MRI-based neuroimaging |

# Palaeontology

**Specimen provenance**

The bradoriids were collected from the Yu'anshan Member, Chiungchussu Formation, Eoredlichia-Wutingaspis trilobite biozone, Cambrian Series 2, Stage 3, Yunnan Province, China. Two specimens of Km. douvillei (YKLP 16233, YKLP 16235) were collected from Mafang, Haikou, Kunming; one specimen of Ky. cheni (YKLP 16232) is preserved on the same rock slab with Km. douvillei YKLP 16233. A second Ky. cheni (YKLP 16236) was collected from Erjie, Jinning, Kunming. The specimen of Indiana sp. (YKLP 16231) was collected from Mt. Jianshan, Haikou. All local permissions were sought and given.

**Specimen deposition**

Yunnan Key Laboratory for Palaeobiology, Yunnan University. Specimens are available for study to all bona fide researchers.

**Dating methods**

No dating procedures. Dates are based on existing biostratigraphical parameters.

☒ Tick this box to confirm that the raw and calibrated dates are available in the paper or in Supplementary Information.
